# *Cryptococcus bacillisporus* causing cryptococcoma of the beak of an African grey parrot (*Psittacus erithacus*), Portugal

**DOI:** 10.1016/j.mmcr.2021.08.006

**Published:** 2021-09-03

**Authors:** Carolina Silva, Carles Juan-Sallés, Joana Mendes, Ana Mendes, Mariana Ruivo, Juan L. Abad, Ferry Hagen, Maria F. Colom

**Affiliations:** a VetExóticos, Clínica Veterinaria, Almada, Portugal; b Noah's Path. Veterinary Pathology Laboratory Specialising in Wildlife and Exotic Species, Elche, Spain; c Medical Mycology Laboratory, University Miguel Hernández, Sant Joan D’Alacant, Spain; d Westerdijk Fungal Biodiversity Institute, Utrecht, the Netherlands; e Department of Medical Microbiology, University Medical Center Utrecht, Utrecht, the Netherlands; f Institute for Healthcare and Biomedical Research of Alicante, ISABIAL, Alicante, Spain

**Keywords:** *Cryptococcus bacillisporus*, African grey parrot, *Psittacus erithacus*, AFLP5/VGIII

## Abstract

We report a severe case of rhinothecal cryptococcoma in a 13-year-old female African Grey Parrot (*Psittacus erithacus*). The bird was born and bred in captivity in Portugal. The beak was deformed and showed several round soft masses, and microscopic examination revealed the presence of cells suggestive for *Cryptococcus*. Phenotypic and molecular analyses identified the obtained yeast culture as *C. bacillisporus* (AFLP5/VGIII). By phylogenetic analysis the parrot strain clustered with clinical *C. bacillisporus* strains from Mexico.

## 1 Introduction

Cryptococcosis is a worldwide-distributed fungal disease affecting humans and animals most commonly caused by members of the *Cryptococcus neoformans* and *Cryptococcus gattii* species complexes that have been reported to infect a wide range of species, including domestic pets, livestock, and wildlife species.

There is an extensive literature concerning the relation between birds and the excretion of *Cryptococcus* spp., especially in pigeons [1,2]. However, there are few reports of clinical cryptococcosis in birds. Their higher body temperature (around 40 °C in almost all birds, except ratites) is thought to inhibit *Cryptococcus* spp. growth and therefore makes it more difficult for this microorganism to cause disease in birds. Among the cases of avian cryptococcosis reported, the most common species affected are psittacines and columbiformes [3]. In 2003, Malik et al. [3] described two main presentations of clinical cryptococcosis in parrots. One of which consists of a locally invasive infection, normally limited to the beak and the upper respiratory tract, mainly diagnosed in Australia; the other consists of a more severe infection of either the lower respiratory tract or systemic involvement, more commonly seen in America's [[3], [4], [5]]. In Europe, there have been several reports of severe systemic cryptococcosis in pigeons, but not in psittacine species. In this report, we describe a case of a local invasive cryptococcal infection affecting the rhinotheca of an African Grey Parrot (*Psittacus erithacus*) born and bred in Portugal, Europe.

## 2 Case

**Fig. 1:**
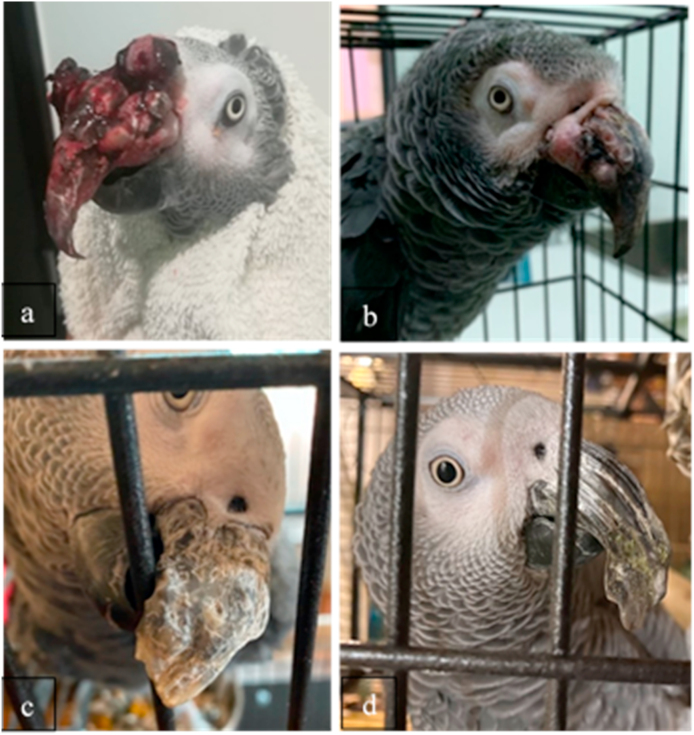
External aspect of the beak lesions on the African Grey Parrot: a) Day of the first consultation. Several round masses and acute active haemorrhage. b) Beak lesions after 10 days of hospitalization. c) Beak lesions after a month of oral treatment at home (note that the fungal masses have almost disappeared, but chronic lesions remain, making the beak surface irregular and deformed, with whitish discolouration). d) Beak lesions after two years of the beginning of the process. Note that the proximal rhinotheca surface is closer to normal shape and colour, while the distal, overgrown part is still deformed and overgrown. (For interpretation of the references to colour in this figure legend, the reader is referred to the Web version of this article.)

A 13-year-old female African Grey Parrot (*Psittacus erithacus*) was brought to the veterinary practice VetExóticos (Almada, Portugal) due to an acute episode of haemorrhage triggered by an excessive scratching of the beak. At the time of presentation, the patient showed an overgrown and deformed beak, with several bleeding soft round masses located in the dorsal and lateral areas of the rhinotheca (Fig. 1a). Another round shape and firm mass was identified on the right wing. During the consultation, the bird was bright and active and showed a good body condition. No other alterations were observed during the physical examination.

According to the owners, this parrot had been bought 13 years ago as a chick and was hand-fed until weaning. It had not lived with any other pets, and it had never left the household. The general external aspect of the beak had been irregular and slightly deformed since they got the parrot, but the masses appeared approximately 8 months before and increased in size during the previous two months. The mass on the wing had appeared several years earlier, but they had not noticed any additional growth. The owners did not report any other current or previous medical condition. The patient was active prior to the bleeding, and it was eating normally.

During the clinical examination bleeding was stopped with nitrate powder and adrenaline-soaked cotton swabs. The patient was hospitalized to start the diagnostic and treatment procedures. Initially, she was administered a combination of meloxicam 1mg/kg IM every 12 hours, enrofloxacin 5 mg/kg IM every 12 hours, tramadol 7 mg/kg SC BID and syringe-forced feeding depending on the patient's voluntary food intake.

The bird remained hospitalized for 10 days until bleeding was controlled and lesions improved. At day 10 of hospitalization and given the suspicion of an underlying neoplastic disease, she was sedated with inhaled isoflurane 1.5% and a biopsy of one of the masses was obtained. Examination and diagnostic tests of the wing's mass were not authorized by the owners. The sample from the beak was sent for histopathologic examination to Noah's Path Laboratory (Spain). After 12 days of hospitalization, the patient was discharged. The beak was still overgrown and deformed, but bleeding had been controlled and the wounds caused by scratching were completely healed, as shown in Fig. 1b.

**Fig. 2:**
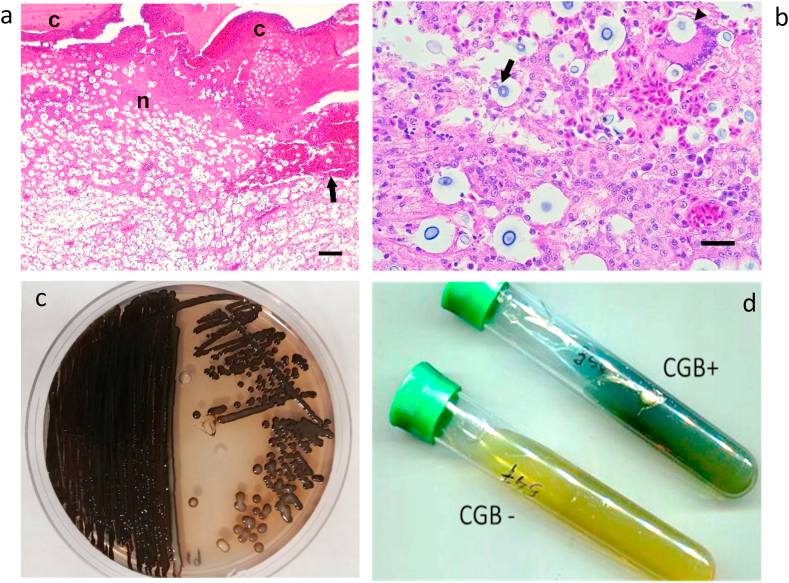
Histopathology and microbiological tests. a) Note necrosis (n) and haemorrhage (arrow) with serocellular crusts (c) covering this lesion. The dermal lesions and crusts contain abundant yeast-like cells surrounded by a thick clear halo. Bar = 180 μm. b) Diffuse infiltration of macrophages around yeast-like cells with a thick clear halo and occasional narrow-based budding (arrow). Note the presence of a multinucleate giant cell that contains a yeast-like cell within its cytoplasm. Bar = 70 μm. c) Phenotypic identification of the yeast by means of melanoid pigment synthesis in l-DOPA agar after 5 days of incubation and d) growth in CGB medium with characteristic dark blue pigment. (For interpretation of the references to colour in this figure legend, the reader is referred to the Web version of this article.)

Histopathological examination revealed severe chronic granulomatous and necrotising dermatitis with intralesional yeast-like cells. Dermatitis was characterized by areas of necrosis with haemorrhage covered by thick sero-cellular crusts (Fig. 2a) and hyperplastic epidermis, and partly surrounded by dense fibrous tissue. Additionally, foci of diffuse infiltration of macrophages and multinucleate giant cells were also noted (Fig. 2b). Both the necrotic and granulomatous lesions contained abundant round to elliptical yeast-like cells surrounded by a thick clear halo, with an approximate total diameter ranging from 15 to 80 μm. Narrow based budding was observed in some of these cells. This yeast cell morphology was characteristic of cryptococcosis. Based on this observation, treatment was modified, and fluconazole was added with a dose of 50 mg per litre of drinking water, and daily water change. An additional sample from the mass was obtained and sent to the Medical Mycology laboratory at Miguel Hernández University (Alicante, Spain) to confirm cryptococcosis and to attempt fungal species identification. The sample was cultured onto Sabouraud Dextrose Agar (SDA) and l-DOPA Agar and incubated at 30 °C. After 48 hours incubation, white mucous colonies developed in SDA and L-DOPA agar. The latter appeared white and smooth after 72 hours of incubation turning slowly into dark brown coloured colonies in 5 days (Fig. 2c).

**Fig. 3:**
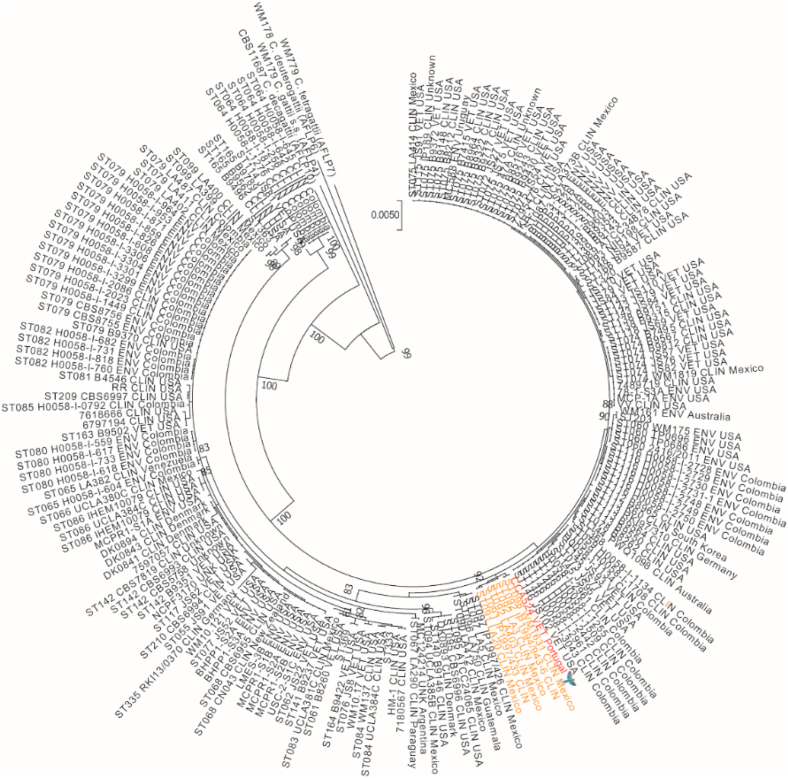
Bootstrapped (1000 × ) maximum likelihood phylogenetic tree analysis based on multi-locus sequence typing data of the veterinary *C. bacillisporus* strain from Portugal, compared to a global set of *C. bacillisporus* strains [[8], [9], [10], [11]]. The veterinary strain from Portugal (indicated in red + with a parrot icon) clustered together with strains from Mexico (orange). (For interpretation of the references to colour in this figure legend, the reader is referred to the Web version of this article.)

The yeast cells obtained from the cultures were further studied by means of phenotypic and molecular analysis. Additionally, antifungal susceptibility for amphotericin B, fluconazole and voriconazole was tested by means of E-test strips. Urea hydrolysis was positive, as well as growth at 37 °C on SDA. Negative staining with Indian ink showed thin capsulated budding yeasts. Assimilation of carbohydrates was tested by Auxacolor2 (BioRad®), showing the typical profile corresponding to members of the *Cryptococcus gattii* and *C. neoformans* species complexes. The yeast was able to grow on Canavanine Glycine Bromothymol Blue (CGB) medium onto which a blue colour appeared after the growth of the yeast cells (Fig. 2d). All these phenotypic features allowed for the identification of the *Cryptococcus gattii sensu lato*. DNA extraction from cultured cells was performed by means of Instagene matrix (BioRad®) and the *URA5* gene was amplified and subsequently digested by HhaI and Cfr13I restriction enzymes [6]. The RFLP fingerprinting obtained corresponded to molecular type VGIII, meaning that it was likely a *C. bacillisporus* AFLP5/VGIII. For further molecular analysis, cryptococcal cells were sent to the Westerdijk Fungal Biodiversity Institute (Utrecht, The Netherlands) where they were subjected to Multi-Locus Sequence Typing (MLST) following the guidelines for molecular typing of pathogenic *Cryptococcus* [7] and compared to MLST profiles of previous studies [[8], [9], [10], [11]] pursuant to the procedure previously described [8]. A 1000 × bootstrapped maximum likelihood phylogenetic analysis was performed using MEGA v7 with the settings for the model ‘Hasegawa-Kishino-Yano’, rates and patterns ‘Gamma distributed with Invariant sites (G + I)’ and the heuristic method ‘Nearest-Neighbor-Interchange (NNI)’. This showed that the parrot strain clustered together with *C. bacillisporus* strains from Mexico (Fig. 3). The strain was deposited in the CBS yeast culture collection (hosted at the Westerdijk Fungal Biodiversity Institute) with accession number CBS16379 (=CCA524; 2MG3508). MLST data were deposited in the NCBI GenBank with accession numbers MW418628-MW418634. Antifungal susceptibility testing indicated a low response to fluconazole; therefore, treatment was switched to amphotericin B 10% oral suspension that was added to the parrot's drinking water (600 mg per litre) and was daily refreshed. This antifungal treatment resulted in good response after 6 months. The size of the mass was significantly reduced, although some chronic lesions remained in the rhinotheca, which clinically seemed to consist mainly of keratin deposition anomalies and deformed beak growth (Fig. 1c).

Pet owners continued reporting to the practice, but they did not bring their parrot for a follow-up. According to them, her condition and the fungal lesions improved after the administration of amphotericin B, but the lesions did not fully heal. Two years after the initial presentation a significant improvement of the lesions and beak deformation were seen, as depicted by photo's provided by the owners (Fig. 1d). So far, haemorrhage was not reported.

## 3 Discussion

The basidiomycetous genus *Cryptococcus* contains ten species; the pathogenic species that are part of the *C. gattii* and *C. neoformans* species complexes are the ones most commonly involved in disease in humans and animals, including birds [4]. *Cryptococcus neoformans* has a worldwide distribution. It is considered an opportunistic pathogen in both humans and animals, and it has been most commonly reported as the cause of systemic cryptococcosis in psittacine species [4]. However, it has also been reported sporadically as the cause of granulomatous lesions in the face, beak, and sinuses in a range of psittacine birds, most notably eclectus parrots (*Eclectus roratus)*, African grey parrots (*Psittacus erithacus*) and macaws (*Ara* spp.) [12].

*Cryptococcus gattii sensu lato* infections in psittacines are mostly reported in Australia; however, several cases have been reported across the American continent [4,5,13]. Few species in this complex are considered as primary pathogens, affecting immunocompetent hosts and causing local lesions. *Cryptococcus bacillisporus* is an extraordinary species within the *C. gattii* species complex as it contains two subtypes (AFLP5A/VGIIIa and AFLP5B/VGIIIb) that have different serotypes (B and C). Besides, this species has a strong association with immunocompromised hosts, while there seems to be a significant difference in disease presentation for both subtypes [9]. In birds, the most common clinical presentation consists of proliferative, fleshy, ulcerated lesions in the upper beak or nasal cavity [4]. In some cases, the local infection can spread to other organs and result in a disseminated cryptococcosis [4,5].

*Cryptococcus bacillisporus* is a relatively rare cause of cryptococcosis in humans and animals, and most cases have been described in warm areas of the Americas and Oceania [11,14]. Its description as a cause of cryptococcosis is exceptional in Europe [8]. In this case, we described a severe rhinothecal cryptococcosis caused by *C. bacillisporus* in a parrot that, according to the pet owners, was born and bred in Portugal. This parrot presented with characteristic beak lesions, consisting of proliferative fleshy masses in the beak, similar to previous reports[[3], [4], [5],^15^]. The combination of surgical debridement and medical antifungal treatment showed good results as reported in previous cases of local cryptococcosis [3].

According to available literature, this is the first apparent autochthonous case of cryptococcosis by *C. bacillisporus* in a psittacine bird from Europe. The environmental setting of the bird has not been studied, so it is difficult to ascertain the origin of this yeast strain. In any case, the alleged lack of contact of this patient with other animals from areas where *C. bacillisporus* is more prevalent, leads us to believe that this may be a more cosmopolitan *Cryptococcus* species than is apparent from clinical data. An exhaustive study of the strains obtained from cases of cryptococcal disease is necessary to obtain more comprehensive knowledge of the geographical distribution of this species.

## Ethical form

A formal written consent to publish the case report signed by the owners of the parrot have been obtained and included in the article submission.

## Declaration of competing interest

The work did not receive any financial support and the authors declared not to have any conflict of interest.
